# Recent Advances in AIEgens for Metal Ion Biosensing and Bioimaging

**DOI:** 10.3390/molecules24244593

**Published:** 2019-12-16

**Authors:** Yongming Li, Huifei Zhong, Yanyan Huang, Rui Zhao

**Affiliations:** 1 Beijing National Laboratory for Molecular Sciences, CAS Key Laboratory of Analytical Chemistry for Living Biosystems, CAS Research/Education Center for Excellence in Molecular Sciences, Institute of Chemistry, Chinese Academy of Sciences, Beijing 100190, China; liyongming@iccas.ac.cn (Y.L.); zhonghuifei@iccas.ac.cn (H.Z.); zhaorui@iccas.ac.cn (R.Z.); 2 University of Chinese Academy of Sciences, Beijing 100049, China

**Keywords:** metal ions, aggregation-induced emission, biosensing, bioimaging

## Abstract

Metal ions play important roles in biological system. Approaches capable of selective and sensitive detection of metal ions in living biosystems provide in situ information and have attracted remarkable research attentions. Among these, fluorescence probes with aggregation-induced emission (AIE) behavior offer unique properties. A variety of AIE fluorogens (AIEgens) have been developed in the past decades for tracing metal ions. This review highlights recent advances (since 2015) in AIE-based sensors for detecting metal ions in biological systems. Major concerns will be devoted to the design principles, sensing performance, and bioimaging applications.

## 1. Introduction

Metal ions play crucial roles in various biological processes and are required by all life forms. The diverse functions of metal ions include osmotic maintenance, signal transduction, catalysis, and proliferation [1]. In addition to functionality, the biodistribution, dynamic ranges, and existing forms of metal ions are also highly varied. Alkali and alkaline earth metal ions are of millimolar level and ubiquitous in every organ. Transition metal ions are usually with lower abundance [2,3]. Owing to their versatile coordination ability, many transition metal ions act as co-factors of macro biomolecules, such as Fe^2+^ in hemoglobin, Zn^2+^ in zinc finger, and Mn^2+^ in photosystems [4,5]. Despite growing knowledge about metal ions, the distribution, metabolism, and mechanism of actions of many metals are largely uncharacterized [1]. The abnormal homeostasis of metal ions has been revealed to be related to diseases including cancer, neurodegenerative disease, and diabetes [6,7]. The exposure and accumulation of toxic metal ions also bring great threat to human health [8,9,10]. However, their exact roles in disease pathologies still largely remain unclear [7]. To address these, sensing systems capable of identification, quantification, and monitoring of metal ions in living biosystems are urgently demanded.

Fluorescent sensors with high sensitivity, simplicity, and visualization have attracted considerable research interests for analyzing biological events in vitro and in vivo. In the past several decades, fluorescent sensors became powerful tools for investigating metal ions in complicated systems. Different recognition moieties and sensing mechanisms were developed to introduce high specificity and sensitivity [11,12,13,14,15]. As an intrinsic property of metal ions, coordination lays an important foundation for metal ion recognition. Reaction-based mechanism is also adopted for selectively sensing respective ions [16,17]. However, conventional fluorescent dyes still suffer from problems including severe background interference, aggregation-induced quenching (ACQ) effect, and poor photostability, which hinder detection sensitivity, real-time and long-term monitoring of biosystems [18,19].

**Figure 1:**
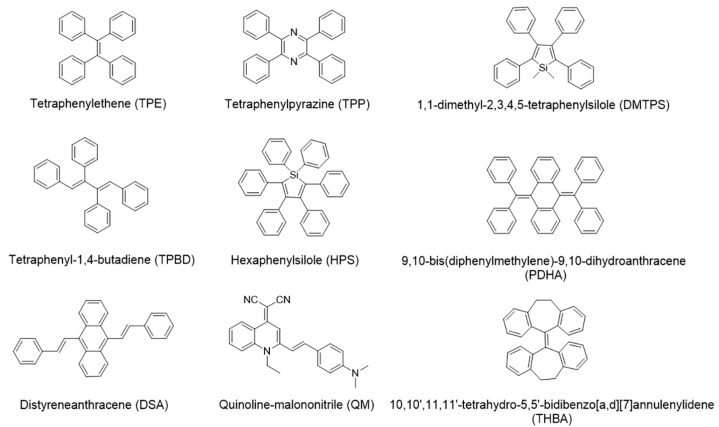
Structure of some common aggregation-induced emission fluorogens (AIEgens).

Aggregation-induced emission fluorogens (AIEgens) first reported by Prof. Tang’s group in 2001 possess unique advantages of activatable emission, good photostability, and compatibility with high concentration thus high sensitivity [20]. These fluorophores are non-emissive or weakly emissive in diluted solution but strongly emissive upon aggregation resulted from the restriction of intramolecular motion (RIM) [21], providing large Stokes’ shift, strong light tolerance and high signal-to-noise ratio [22,23,24,25]. A number of molecules have been reported with AIE behavior, including tetraphenylethene (TPE), hexaphenylsilole (HPS), and quinoline-malononitrile (QM) [19,22,23] (Figure 1). Their tailorable structures allow further modification of the AIEgens with functional molecules and materials, such as nucleic acids, peptides, graphene, and metal-organic frameworks (MOFs) [26,27,28,29]. The successful applications have been demonstrated in the fields of detection, disease diagnosis, and therapy [30,31,32,33,34]. In terms of metal ion analysis, different AIE-based sensing systems have been developed which accelerate the understanding the roles of these ions in biological samples. The sensing mechanism is crucial for the analytical performance of the sensors. These systems usually contain two parts: recognition moiety and signaling moiety. AIE cores play the role of signal output, while modifications on AIE structures enable the selective recognition. Coordination chemistry and metal-related reactions are two major approaches for the recognition. The presence of target ion changes the signal output of AIE cores, which enables sensitive detection.

Given the importance of metal ions and the continuously growing body of AIEgens for metal ion detection, this review focuses on recent development of AIE-based sensors for detecting and imaging of metal ions in biological systems. Major concerns will be devoted to the design principles, sensing performance, and applications for tracing metal ions in vitro and in vivo.

## 2. Alkali Metal Ions

Alkali metals include lithium (Li), sodium (Na), potassium (K), rubidium (Rb), cesium (Cs), and francium (Fr). The concentration of Na^+^ and K^+^ in human body is of millimolar level and differs widely between intracellular and extracellular region [3]. Crown ether, aza-crown ether, and their derivates are the traditional recognition components for these ions due to the selection of cavity. The sensing and detection of alkali metal ions are mainly focus on Li^+^, Na^+^, and K^+^. There has been an excellent review on fluorescent probes for alkali metals and alkaline earth metals [35].

For conventional AIEgens, water solubility is usually a problem which prevents their applications for biosensing and bioimaging. The introduction of biomolecules can overcome this issue. Tan et al. modified TPE derivative with G-rich DNA oligonucleotide for the detection and imaging of K^+^ [36]. The oligonucleotide moiety not only enhanced water solubility, but also provided biocompatibility and cell permeability. This probe was weakly emissive in aqueous solution. In the presence of K^+^, parallel G-Quadruplex structure could be induced, which gathered TPE groups. The π-π stacking interactions among phenyl-ring motifs of TPE derivatives further stabilized the G-Quadruplex structure. Thereby, fluorescent emission was significantly enhanced. This turn-on probe showed a detection limit of 5 μM towards K^+^ in Tris-HCl buffer. The feasibility of the probe for sensing K^+^ in living cells was also demonstrated. Strong fluorescence was emitted from cytoplasm of HeLa cells after treated with the probe. The disappearance of the blue emission after the treatment K^+^ suppressing reagents confirmed the feasibility and reversibility of this probe for detection and imaging of K^+^.

## 3. Alkaline Earth Metal Ions

Alkaline earth metals are in group 2 of the periodic table, including beryllium (Be), magnesium (Mg), calcium (Ca), strontium (Sr), barium (Ba), and radium (Ra). Among these metals, Mg and Ca are involved in many biological processes, such as signal transduction and regulation of enzyme activity. The normal concentrations of Mg^2+^ and Ca^2+^ are usually in the ranges of 0.5–20 mM and 0.1 μM–2 mM, respectively [3]. The abnormality in their concentrations may cause a series of diseases, such as hypertension, osteoporosis, neuronal injury, soft tissue calcification, and hypercalcemia [37,38,39]. Probes containing β-ketoester, β-carboxyester, benzylguanine, or carboxyl moiety are usually designed to detect and sense Mg^2+^ and Ca^2+^ [35].

For AIEgens, carboxyl or nitrogenous groups are usually integrated into the structures for Mg^2+^ and Ca^2+^ binding. Chemoensors including small molecules and polymers based on AIE phenomenon have been developed [40,41,42]. An AIE probe synthesized by the condensation reaction between benzene-1, 2-diamine and 5-bromo-2-hydroxybenzaldehyde was reported for Mg^2+^ detection [40]. A group of polymer sensors based on TPE appended poly(acrylic acid) derivatives [PAA-TPE_x_ (x = 0.01–0.05)] were reported for detecting Ca^2+^ [41,42]. The binding towards Ca^2+^ was realized via the coordination behavior of carboxyl groups. These sensors showed selectivity of Ca^2+^ over Mg^2+^ due to the more effective induction of chain folding.

**Figure 2:**
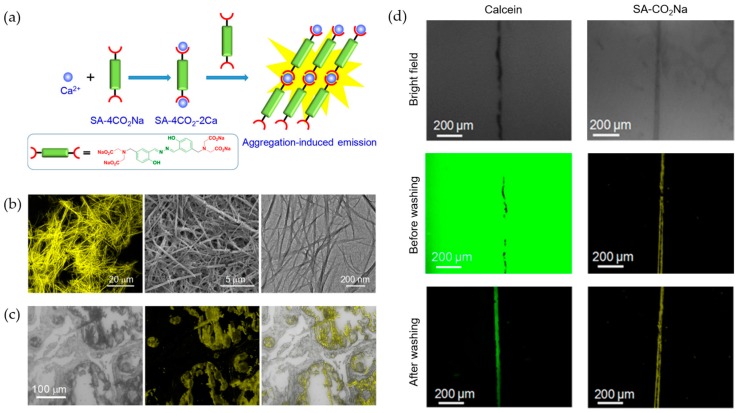
(**a**) Ca^2+^ sensing mechanism of the turn-on probe SA-4CO_2_Na [43] (Reproduced with permission from [43]). (**b**) Fluorescence, SEM, and TEM images of SA-4CO_2_Na upon Ca^2+^ addition. (**c**) Fluorescence images of calcium deposits in psammomatous meningioma slice. (**d**) Fluorescence images of bovine bone microcracks by treated with calcein and SA-4CO_2_Na.

Water solubility, high selectivity and appropriate responding range are important for analyzing Mg^2+^ and Ca^2+^ in living systems. An AIE-active Ca^2+^ probe (SA-4CO_2_Na) was developed with a millimolar responding range which fitted the requirement for Ca^2+^ analysis in biosystems [43]. Two iminodiacetate groups with negative charge were modified to SA fluorogen acting as recognition groups (Figure 2a). In the presence of Ca^2+^, the chelation between two iminodiacetate groups and one metal ion gave fibrillar aggregates with strong fluorescence enhancement at 541 nm (Figure 2b). The linear range of 0.6–3.0 mM was suitable to discriminate normal (1.0–1.4 mM) and hypercalcemic (1.4–3.0 mM) Ca^2+^ concentration. SA-4CO_2_Na was successfully applied to imaging Ca^2+^ in different biological samples, including human psammomatous meningioma slice with calcium deposits and bovine bone microcracks (Figure 2c,d). Compared with commercial dyes calcein, SA-4CO_2_Na could image Ca^2+^ with high signal-to-noise ratio without washing. Selective imaging of Ca^2+^ with clear background demonstrated great potential of this probe for broad biomedical applications.

**Figure 3:**
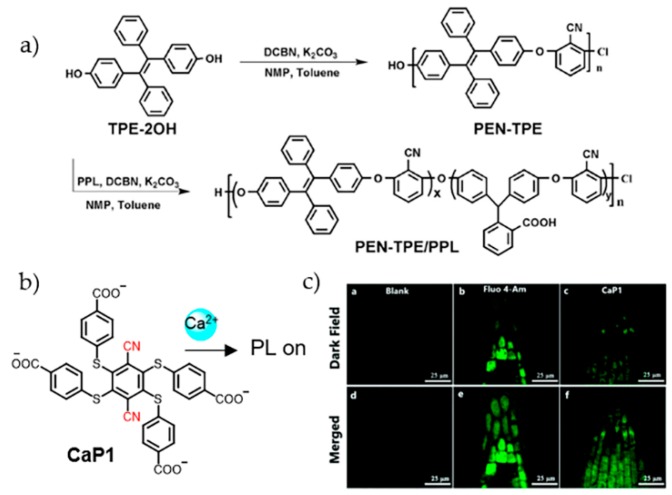
(**a**) Synthesis of a copolymer PEN-TPE/PPL for the detection of Ca^2+^ [45]. (**b**,**c**) A molecular probe (**CaP1**) with Ca^2+^-responsive signal for analyzing Ca^2+^ in the root cells of *Arabidopsis thaliana* [46] (Reproduced with permission from [45,46]).

With water solubility and coordinating ability towards Ca^2+^, bidentate pyridine carboxylate was anchored on TPE for the design of Ca^2+^ sensor [44]. Upon Ca^2+^ recognition, coordination oligomers or polymers formed with decreased solubility. The aggregation enhanced fluorescence emission which released detectable signal. The sensor was applied for imaging Ca^2+^ in A549 cells. The appearance of blue fluorescence in cytoplasmic area suggested the binding of Ca^2+^. AIEgens are also involved in the structures of polymers for the development of ion sensors. Liu et al. synthesized a copolymer PEN-TPE/PPL (PEN: polyarylene ether nitrile; TPE: tetraphenylethene; PPL: phenolphthalin) through the copolymerization of TPE-2OH, 2, 6-dichlorobenzonitile (DCBN) and phenolphthalein (PPL) (Figure 3a) [45]. With the presence of carboxyl group, PEN-TPE/PPL showed coordination towards metal ions, including Cu^2+^, Pb^2+^, Zn^2+^, and Ca^2+^. Among these, Ca^2+^ caused the largest emission enhancement. Mechanism investigation indicated that Ca^2+^ induced crosslinking of PEN-TPE/PPL and the formation of nanospheres. These nanospheres could penetrate EMT-6 cells and concentrate in cytoplasm.

Aggregation-induced phosphorescence (AIP) property was also introduced for the detection of Ca^2+^. Recently, a molecular probe (**CaP1**) with phosphorescent properties was developed [46]. In the presence of Ca^2+^, each **CaP1** molecule coordinated two Ca^2+^ ions via cyano and carboxyl groups to form linear long chains (Figure 3b). Precipitated particles were produced with AIP characteristics. Due to the long lifetime of phosphorescence, time-gated detection method was established which effectively inhibited autofluorescence. **CaP1** was observed to successfully enter cells and emit phosphorescence responding to Ca^2+^ in the root cells of *Arabidopsis thaliana*, which could be a useful tool for studying the biological roles of Ca^2+^ in plants (Figure 3c).

## 4. Transition Metal Ions

Transition metals include a large family of metal elements. These metals vary in chemical properties, concentration, distribution, and acting roles in living biosystems. Most transition metal ions show good coordination ability, which is one main point to design binding group. Metal-mediated reactions are also useful to selectively recognize metal ions [16,17,47,48]. In this part, several common transition metal ions sensed by AIE probes are discussed, including Mercury, Fe^3+^, Cu^2+^, Zn^2+^, Ag^+^, Ni^2+^, Cr^3+^, and Au^3+^.

### 4.1. Mercury Ions

Mercury is toxic and a great threat to human health. The bioaccumulation of mercury can cause severe damage to many organs, such as central nervous system, lung, and kidneys [49,50]. Different mechanisms have been investigated for specific sensing of mercury based on AIE phenomenon.

Coordination chemistry has been employed for the recognition of Hg^2+^ [51,52,53,54,55,56,57,58,59,60]. Structures containing Schiff base, sulfydryl, and imidazole groups show effectiveness for Hg^2+^ binding. Huo et al. synthesized two probes by decorating diaminomaleonitrile moiety on TPE [51]. These probes formed nanoscale aggregates and were emissive in solution with high water fraction (*f*_w_). In the presence of Hg^2+^, the N atoms in diaminomaleonitrile moiety bound Hg^2+^, which disturbed internal charge transfer and caused fluorescence quenching. This turn-off response was applied for detecting Hg^2+^ in living cells. Considering the complexity of living systems, turn-on probes can provide accurate information. By incorporating Schiff base unit, Yang et al. designed and synthesized a series of α-cyanostilbene derivatives for Hg^2+^ detection [52,53,54]. Hg^2+^ binding drove π-π stacking and hydrogen bonding between adjacent probes, which hampered the intramolecular rotation thus caused fluorescence enhancement. With improved emission performance, two of these probes were used for imaging Hg^2+^ in living cells. Recently, a ratiometric probe using an AIEgen has been used for the imaging of Hg^2+^ in onion inner and outer epidermal tissues [61].

**Figure 4:**
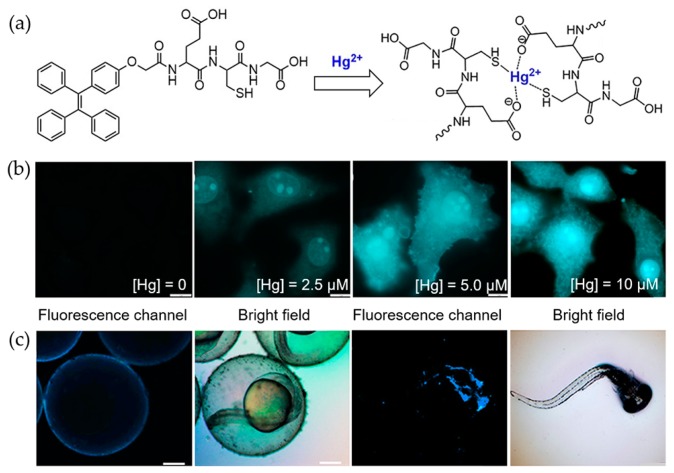
Bind mechanism and sensing performance of peptide-based probe for Hg^2+^ [55] (Reproduced with permission from [55]). (**a**) Structure of the probe and binding mechanism to Hg^2+^. (**b**) Fluorescence imaging in HeLa cells after incubation with probe and different concentrations of Hg^2+^. (**c**) Fluorescence imaging in zebrafish embryo and zebrafish larvae after treated with Hg^2+^ and probe.

In addition to detection, tracing biological distribution of Hg^2+^ is important for studying its toxicology. A peptide-based probe was designed by using a tripeptide as Hg^2+^-targeting moiety and TPE as the signaling group [55]. The incorporation of the peptide not only allowed the highly specific turn-on discrimination of Hg^2+^ from 19 different metal ions, but also provided high compatibility with physiological environment. For mechanism investigation, peptide analogues were also conjugated with TPE, which suggested the thiol and carboxyl side chains played important roles for the binding with Hg^2+^ (Figure 4a). Further applications of the sensor for monitoring Hg^2+^ in living cells and zebrafish were demonstrated. The observation that Hg^2+^ showed high tendency to accumulate in cell nucleus indicated the damage effect of this ion to this subcellular compartment (Figure 4b). As indicated by the signal of the sensor, the distribution of Hg^2+^ in chorion of zebrafish embryo and the brain of larvae was related to the deleterious effect of inorganic mercury in living biosystems (Figure 4c).

**Figure 5:**
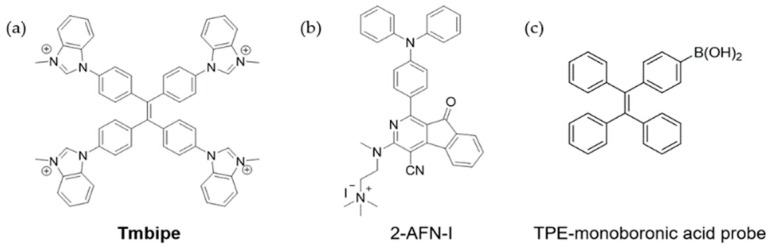
Molecular structures of chemosensors for mercury ions. (**a**) **Tmbipe** [56]. (**b**) 2-AFN-I [57]. (**c**) A TPE-monoboronic acid probe [64] (Reproduced with permission from [56,57,64]).

The bioaccumulation of Hg^2+^ leads to the formation to highly toxic organomercury compounds such as methylmercury (MeHg^+^) and phenylmercury (PhHg^+^). Therefore, detection of both inorganic and organic species is crucial to protect ecosystem and human health. Recently, Kong et al. modified four methylated benzimidazole groups on the TPE skeleton to prepare a water soluble AIE probe **Tmbipe** (Figure 5a) [56]. In addition to Hg^2+^, benzimidazole groups in **Tmbipe** could also bind MeHg^+^ and PhHg^+^, producing Hg^2+^-tetracarbene complex via C-Hg bonds. The formation of chelate ring restricted the intramolecular rotation and increased molecular planarity, which induced the formation of aggregates and further restricted the molecule skeletal vibration. Due to the unusual coordination mode of C-Hg, this probe showed high selectivity towards Hg^2+^ and organomercury. The detection limits for Hg^2+^, MeHg^+^, and PhHg^+^ were estimated as 63 nM, 94 nM, and 78 nM, respectively. With good solubility, **Tmbipe** was successfully applied for imaging Hg^2+^ in different cells. In another work, a dual detection strategy for the bioaccumulation of Hg^2+^ in *P. phosphoreum* was developed by using an AIE sensor 2-AFN-I (Figure 5b) [57]. Hg^2+^ quenched the strong bioluminescence of *P. phosphoreum* by interrupting quorum sensing system, meanwhile increased the emission of 2-AFN-I inside the bacteria. This strategy provided a useful inspiration for the imaging and evaluation of bioaccumulated toxins in *P. phosphoreum*.

In addition to coordination chemistry strategy, reaction-based mechanism is another effective way to sense Hg^2+^ [62,63]. Joshi et al. designed a TPE-monoboronic acid probe to sense and image Hg^2+^ and CH_3_Hg^+^ based on mercury ion-promoted transmetalation reaction (Figure 5c) [64]. After reaction, the transformation of the C-B bond in the probe to C-Hg gave poorly soluble TPE-HgCl or TPE-HgMe product. The resultant aggregates, restriction of intramolecular rotation caused fluorescence enhancement. This probe was used to image methylmercury in HEK cells and zebrafish. For CH_3_HgCl pretreated samples, strong blue emission was observed in cells and the whole body of zebrafish. In other examples, Hg^2+^-induced umpolung reaction was reported to sensing Hg^2+^ [65,66]. 2-mercaptoethanol was integrated with the AIEgen to increase solubility and provide dethioacetalization site. In the presence of Hg^2+^, mercaptoethanol was substituted by an aldehyde group, leading to AIE phenomenon. Further applications in the detection of Hg^2+^ in river water, urine samples, living cells, and zebrafish were achieved.

Considering the co-existence of multiple species in complicated biosamples, multi-targeting sensors were developed with AIE behaviors. Some dual-responsive strategies have been reported to sense Hg^2+^ and other targets [67,68]. By combining AIE organic nanoparticles with Au nanoparticles, Ouyang et al. developed a dual-emission fluorescent sensor to detect mercury and melamine [68]. The composite exhibited maximum emission wavelengths at 525 nm and 625 nm, respectively, under excitation at 365 nm. In the present of Hg^2+^, the red fluorescence of Au NPs was quenched via metallophilic Hg^2+^-Au interactions while the green emission of the AIE particles almost remained unchanged, generating a ratiometric fluorescent signal. With higher affinity for Hg^2+^, melamine prevented the fluorescence quenching thus also could be detected with this sensor. Cell imaging assays demonstrated the effectiveness of this sensing strategy.

### 4.2. Cu^2+^

Copper, as an important trace element, participates in the formation of some enzymes and proteins, such as amine oxidase and ceruloplasmin. However, high concentration of Cu^2+^ is toxic [69]. Derivates based on rhodamine are frequently used to detect Cu^2+^ via Cu^2+^-induced spirolactam ring-opening and hydrolysis processes to produce a turn-on response [70,71]. In the past years, AIE-based probes have also been designed for Cu^2+^ sensing and find applications in bioimaging [72,73,74,75].

**Figure 6:**
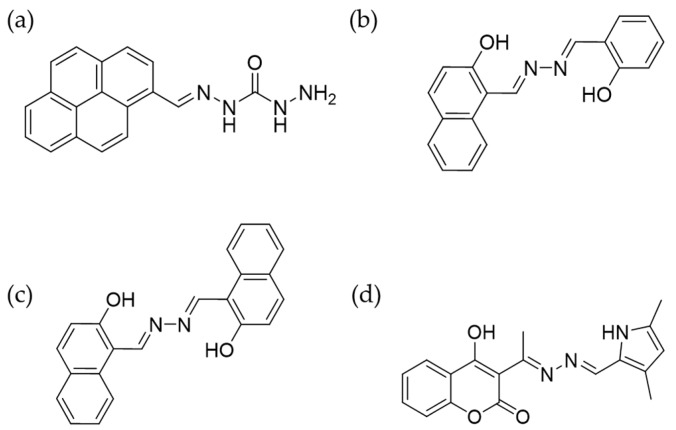
Molecular strucutres of chemosensor for Cu^2+^. Cu^2+^ sensors reported in [72] (**a**), [73] (**b**), [73] (**c**), and [74] (**d**) (Reproduced with permission from [72,73,74]).

Based on the coordination behavior of Schiff base towards Cu^2+^, Hou et al. synthesized a Schiff base derivative with a Cu^2+^ binding stoichiometry of 2:1 (Figure 6a) [72]. The resultant fluorescence enhancement at 455 nm was used as the signal for Cu^2+^ detection in HeLa cells. In addition to turn-on sensors, turn-off strategy was also employed for the development of Cu^2+^ probes by using Schiff base as the core structure. The condensation of salicylaldehyde and 2-hydroxy-1-naphthaldehyde presented two probes with Schiff base moiety (Figure 6b,c) [73]. These two probes were highly emissive at 534 nm and 530 nm, respectively, by forming micron particles in water. The coordination with Cu^2+^ broke the planar conformation of the probes, therefore quenched the fluorescence. The turn-off signal was used to indicate the appearance of Cu^2+^ in KYSE510 cells. Another probe with pyrrole and coumarin units connected by Schiff base was also reported to detect Cu^2+^ by fluorescence quenching (Figure 6d) [74]. This probe displayed different emission in solvents with varied water/DMSO fractions. Fluorescence was quenched upon Cu^2+^ addition due to photoinduced electron transfer (PET) process and disassembling of the aggregates. The sensor was utilized for Cu^2+^ imaging in HeLa cells.

He et al. reported a dual-detection strategy for Cu^2+^ and ATP [75]. An amphiphile probe was designed by modifying oxyalkyl chains with 1,5,9-triazacyclododecane unit as Cu^2+^-targeting group. This probe assembled into micelles and emitted at 491 nm in aqueous solution. Upon Cu^2+^ chelation, fluorescence was quenched. Since ATP has higher affinity with Cu^2+^, fluorescence can be recovered. Such fluorescence recovery responded specifically to ATP with slightly interference from ADP. The on-off-on mode provided detection limits of 0.1 μM for Cu^2+^ and 1.5 μM for ATP. The quenching and recovering of blue emission located in cytoplasm of HeLa cells was successfully achieved.

### 4.3. Zn^2+^

Zn^2+^ plays essential roles in the formation of zinc finger proteins, enzyme catalysis and signaling. The intracellular concentration of Zn^2+^ is around 200 μM [76]. The imbalance of Zn^2+^ in human bodies is related to many diseases, including growth retardation, Alzheimer’s disease and defects in immune systems [77,78]. AIE-active fluorescence probes have been developed for Zn^2+^ detection and sensing [79,80,81,82,83].

**Figure 7:**
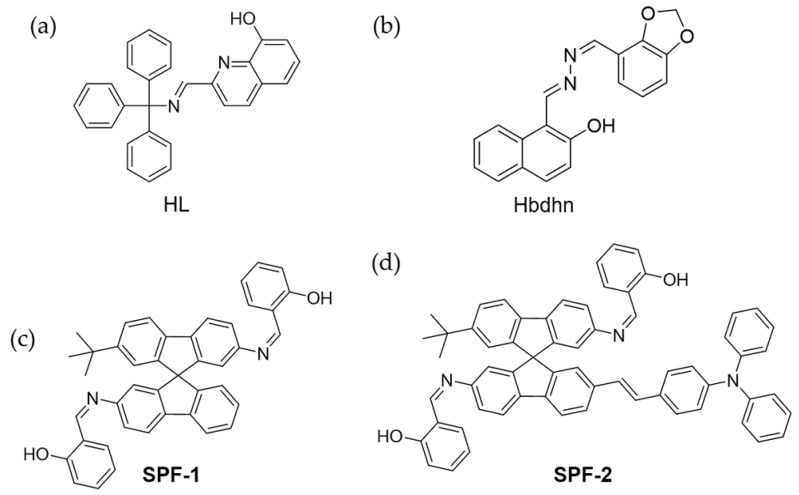
Molecular structures of chemosensors for Zn^2+^. (**a**) 2-(Trityliminomethyl)-quinolin-8-ol (HL) [79]. (**b**) Hbdhn [83]. (**c**) **SPF-1** [81]. (**d**) **SPF-2** [81] (Reproduced with permission from [79,81,83]).

Jin and coworkers designed a Schiff-based compound, 2-(Trityliminomethyl)-quinolin-8-ol (HL), as ligand to coordinate with Zn^2+^ (Figure 7a) [79]. HL was non-emissive in THF/H_2_O solution. However, the complex ZnL_2_ formed nanoscale J-aggregates through coordination bonds and π-π interactions, thus showed obvious fluorescence. This turn-on mode was applied for detecting and imaging Zn^2+^ in SH-SY5Y cells. Recently, another Schiff base chemosensor Hbdhn with AIE properties was developed for imaging Zn^2+^ in living cells. (Figure 7b) [83]. In another example, Fan et al. designed an AIE probe to detect Zn^2+^ and single-stranded DNA (ssDNA) in different solvents [80]. In H_2_O/DMSO (*f_w_* = 80%), the coexistence of Zn^2+^ with this probe caused fluorescence enhancement. However, in H_2_O/DMSO (*f_w_* = 99%) a new metal complex L-Zn^2+^ was produced which was weakly emissive. After addition of ssDNA, the metal coordination between ssDNA and Zn^2+^ enhanced the emission. This dual responsive turn-on approach was used to sense intracellular Zn^2+^ and ssDNA. Xiao et al. synthesized two AIE probes (**SPF-1** and **SPF-2**) for Zn^2+^ sensing, by modifying spirobifluorene (Figure 7c,d) [81]. The detection limits were 0.3 μM and 63 nM, respectively, based on AIE enhancement. **SPF-1** was applied in the intracellular Zn^2+^ imaging of A549 cells with green fluorescence. Notably, **SPF-2** was used for two-photon fluorescence imaging due to the donor-π-acceptor type molecule structure. In the cell imaging, yellow fluorescence was emitted from cells under excitation at 800 nm.

Integrating biomolecule into probes is an effective strategy for biosensing and bioimaging due to the good biocompatibility. Wang et al. designed a peptide-modified TPE probe to sense Zn^2+^ in a turn-on manner [82]. With a sequence of LHLHLRL, the peptide could selectively recognize Zn^2+^ via histidine residues by mimicking the Zn^2+^ binding site of carbonic anhydrase. Two glycine residues were introduced as spacer to connect the fluorophore and recognition group. The TPE unit provided fluorescence signal under aggregates. The detection of Zn^2+^ was realized in 80% aqueous buffered-ethanol solution (1 mM PBS, pH 7.0), with a detection limit of 18.56 nM. This probe could image the intracellular Zn^2+^ in HeLa cells without internal addition of Zn^2+^. Furthermore, the permeability rate and intracellular concentration change of Zn^2+^ were also measured according to the emission of the probe.

In addition to organic probes, some nanoparticles and metal nanoclusters also show AIE behavior [84], and some of them have been used for Zn^2+^ sensing with the merits of simple preparation, broad excitation range and high photostability [85,86]. For instance, copper nanoclusters (Cu NCs) are frequently used for fluorescence analysis with AIE activity [87,88]. Zhao’s group synthesized glutathione (GSH)-capped Cu NCs with AIE property for sensing Zn^2+^ [86]. Upon the addition of Zn^2+^ in buffered aqueous solution, Cu NCs bound Zn^2+^ via surface groups and electrostatic interaction. The crosslinking between clusters caused aggregation and emission enhancement due to the restriction of vibration, rotation, and torsion of Cu NCs. This light-up imaging of Zn^2+^ was also achieved in MGC-803 cells.

### 4.4. Fe^3+^

Fe^3+^ is one of the most common transition metals in the human body and participates in many biological activities, including oxygen carrying, electron transport, and enzyme catalysis. Nevertheless, the abnormality in Fe^3+^ has been revealed to correlate with many diseases, such as anemia, Parkinson’s syndrome and cancer [89,90]. There have been many reports for Fe^3+^ detection with AIE probes [91,92,93,94].

**Figure 8:**
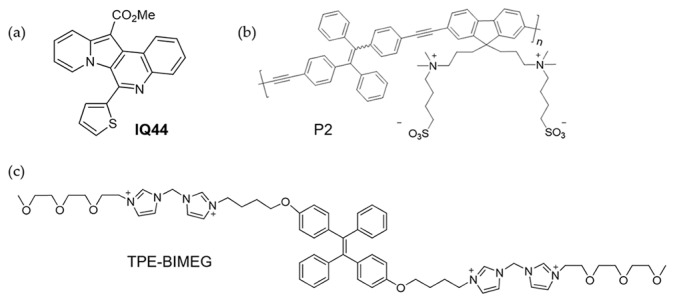
Molecular structures of chemosensors for Fe^3+^. (**a**) **IQ44** [91]. (**b**) P2 [93]. (**c**) TPE-BIMEG [94] (Reproduced with permission from [91,93,94]).

Cyano group is usually used as the recognition group for the coordination of iron ions. Hence, combining cyano group and AIE fluorophore is an effective method to detecting Fe^3+^. Liu et al. designed an AIE probe containing cyano groups and triphenylamine unit to detect Fe^3+^, CN^−^, and SO_3_^2−^ [91]. In aqueous solution (1% DMSO), fluorescence enhancement around 570 nm was observed due to the formation of nanoparticles from the probe. Upon the addition of Fe^3+^, the coordination from Fe^3+^ disturbed the hyperconjugation structure and caused fluorescence quenching. This turn-off mechanism could selectively discriminate Fe^3+^ from Fe^2+^. In imaging experiment, blue fluorescence of the probe was observed in the cytoplasm of HeLa cells. For cells treated with Fe^3+^, blue fluorescence disappeared in cells. Moreover, anion CN^−^ and SO_3_^2−^ also quenched the fluorescence by affecting the charge density and breaking the hyperconjugation structure. Lee et al. reported an iron-selective turn-on sensor **IQ44** which shows high affinity and sensitivity towards Fe^3+^. By localized to lysosomes, **IQ44** can imaging cellular Fe^3+^ in lysosomes, and is promising for studying related biological processes (Figure 8a) [91].

Fluorescence nanoparticles have been explored as sensors to sense Fe^3+^ [93,94]. Wang et al. synthesized a conjugated polymer P2 for sensing Fe^3+^ (Figure 8b) [93]. This polymer containing TPE unit and zwitterionic unit. Then, DSPE-PEG 2000 was reprecipitated with P2, forming lipid-P2 NPs with spherical shape and an average diameter of ~23 nm. Lipid-P2 NPs displayed stable and strong emission at 500 nm in physiological conditions. The appearance of Fe^3+^ could quench the fluorescence. This turn-off response provided a detection limit for Fe^3+^ of 0.22 μM. Bioimaging of Fe^3+^ was achieved in A549 cells. Another example of fluorescent organic nanoparticles via self-assembly to sense and image Fe^3+^ was reported by Li et al. [94]. The fluorophore monomer TPE-BIMEG containing bis-imidazolium (BIM), oligo(ethyleneglycol) (EG), and TPE moieties (Figure 8c) showed good solubility in polar solvents. Due to the interaction between BIM and ATP, TPE-BIMEG could self-assemble into nanoparticles, which exhibited AIE enhancement around 470 nm in aqueous solution. Due to the excited state deactivation effect of Fe^3+^, the fluorescence of the nanoparticles could be quenched by Fe^3+^. The fluorescence quenching at a Fe^3+^ concentration as low as 0.1 nM was observed, suggesting the high sensitivity of the method. During imaging of HeLa cells, strong fluorescence was emitted from the cytoplasm and cell membrane after incubation with TPE-BIMEG. In the presence of Fe^3+^, the measurement of fluorescence at different time points showed the fluorescence in HeLa cells could be completely quenched by Fe^3+^ within 30 min.

### 4.5. Other Transition Metal Ions

Besides the above-mentioned ions, other transition metal ions also draw research attentions.

**Figure 9:**
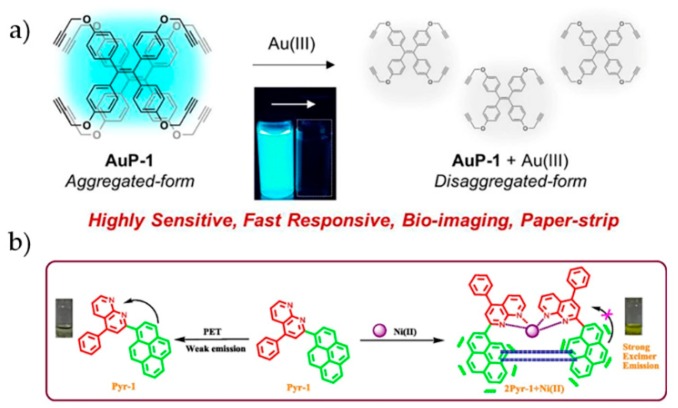
(**a**) Sensing of Au^3+^ with an AIE probe **AuP-1** [96]. (**b**) Pyr-1 for Ni^2+^ sensing [97] (Reproduced with permission from [96,97]).

Hahn et al. utilized TPE bridged tetraimidazolium salts, [H_4_L-Et](PF_6_)_4_, and [H_4_L-Bu](PF_6_)_4_, to chelate Ag^+^ and Au^+^ to form dinuclear tetracarbene complexes [94]. The restriction of the rotation of the phenyl groups caused sharp fluorescence enhancement at 500 nm. Kim et al. designed a TPE probe to detect Au^3+^ based on AIEgen disaggregation effect [95]. The probe **AuP-1** was prepared by decorating the TPE core with four propargyl groups (Figure 9a). In aqueous solution, the fluorescence of **AuP-1** dramatically decreased within 1 min after the addition of Au^3+^. It was proposed that the interaction of Au^3+^ with alkynyl could convert aggregated **AuP-1** to disaggregated form, therefore quenching the fluorescence. With low toxicity and high stability, this probe was applied for imaging Au^3+^ in RAW 264.7 cells. The inhibition of the green fluorescence from **AuP-1** was used as the indicator of Au^3+^ inside cells.

Pitchumani et al. reported a probe **Pyr-1** for Ni^2+^ detection which was synthesized from pyrene and 1,8-naphthyridine units (Figure 9b) [97]. Due to the PET process from pyrene to 1,8-naphthyridine, **Pyr-1** was weakly emissive in dilute solution. Upon the addition of Ni^2+^, obvious fluorescence enhancement at 420 nm and red shift were observed. Mechanism investigation indicated that **Pyr-1** and Ni^2+^ formed a square-planer complex with a stoichiometry of 2:1. The coordination operated between Ni^2+^ and N atoms in 1,8-naphthyridine. The emission enhancement was attributed to the inhibition of PET process and the formation of excimer. This probe showed high selectivity towards Ni^2+^ and a detection limit of 0.25 μM in water. Successful application for Ni^2+^ imaging in HeLa cells was also demonstrated.

## 5. Other Metal Ions

Post-transition metals usually refer to aluminum (Al) gallium (Ga), indium (In), thallium (Tl), tin (Sn), lead (Pb), and bismuth (Bi). Among these metal ions, current reported AIE probes mainly focus on Al^3+^ and Pb^2+^.

**Figure 10:**
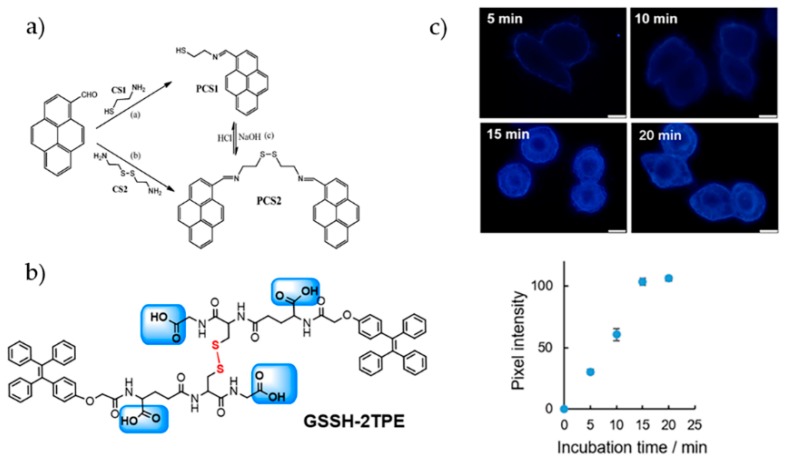
(**a**) Design and synthesis of **PCS1** and **PCS2** [102]. (**b**,**c**) Schematic illustration of GSSH-2TPE for Pb^2+^ sensing and imaging. (**b**) The design and synthesis of GSSH-2TPE. (**c**) Binding kinetics of GSSH-2TPE to Pb^2+^ in HeLa cells [103]. (Reproduced with permission from [102,103]).

Zhao and coworkers designed a simple and effective probe TPE-COOH for Al^3+^ sensing [98]. In the presence of Al^3+^, the formation of coordination complex and nanoaggregates led to activatable emission at 470 nm. The high selectivity was demonstrated by the discrimination of Al^3+^ from various metal ions. Quantitation analysis gave a detection limit of 21.6 nM for Al^3+^. A time-course imaging of Al^3+^ was performed in HeLa cells to record the binding process of TPE-COOH to intracellular Al^3+^. Feng et al. introduced four carboxylate groups into one TPE core to sense Al^3+^ and Pb^2+^ [99]. The probe TPE-4CO_2_Na shows good solubility in pure aqueous solution. Both Al^3+^ and Pb^2+^ can coordinate with TPE-4CO_2_Na to produce millimetre-sized aggregates. To examine the bio-applicability, *Arabidopsis thaliana* were chosen as the model. The acidic form of the probe TPE-4CO_2_H exhibited better cell permeability and could sense the metal ions inside cells. Probes with pyrene structure were also reported to detect Al^3+^, Fe^3+^, and Cr^3+^ [100,101,102]. In one of such examples, monomeric pyrene **PCS1** and dimeric **PCS2** (Figure 10a) displayed AIE characteristic owing to the inhibition of PET/twisted intramolecular charge transfer (TICT) process in aggregates [102]. By using the turn-on response of **PSC1** towards Al^3+^, Fe^3+^, and Cr^3+^, cell imaging experiments with RAW 264.7 cells were performed.

Peptides with tailorable structures and rich coordination chemistry provide rich resource for designing recognition blocks. Huang and coworkers designed a Pb^2+^-specific sensor by mimicking the structure of GSH [103] (Figure 10b). Lewis acid-base theory was employed to guide the modulation of the selectivity of the probe. With both hard Lewis base (the carboxyl group) and soft Lewis base (the thiol group), the leading structure GSH-TPE responded to several metal ions with turn-on fluorescence. After the oxidation of side groups, the probe GSSH-2TPE selectively recognized Pb^2+^ with high affinity due to the matched coordination configuration and cavity size. The complex further assembled into nanoparticles via the intermolecular noncovalent interactions, activating bright fluorescence. Endogenous biothiol species and metal ions such as GSH, cysteine, Mg^2+^, and Ca^2+^ hardly interfered the sensing performance. Cellular binding kinetics and biodistribution of Pb^2+^ were measured (Figure 10c). The higher intensity in cell membrane and cytoplasm suggested stronger retention and accumulation of Pb^2+^ in these compartments.

Uranium belonging to actinides group is a radioactive element and poses great threats to human health. Tang et al. developed a ratiometric fluorescence probe, 3-hydroxy-flavone salicylaldehyde azine (HFSA), for the detection and cell imaging of trace uranyl ion [104]. HFSA showed obvious emission enhancement in water/EtOH (*f_w_* = 80%) at 534 nm due to AIE effect. In the presence of UO_2_^2+^, the emission at 534 nm remained unchanged while a new emission peak appeared at 457 nm. This phenomenon was ascribed to the connection of UO_2_^2+^ with adjacent hydroxy groups in HFSA with a stoichiometric ratio of 1:2. The emission ratio (*I_457_/I_534_*) was linearly dependent on the concentration of UO_2_^2+^ in the range of 0–20 ppb. This probe showed excellent selectivity for UO_2_^2+^ after F^-^ addition and pH adjustment to reduce interference. During cell imaging, only yellow emission was observed in HFSA-treated HeLa cells. For cells loaded with UO_2_^2+^ and HFSA, both yellow and blue fluorescence was emitted, demonstrating the effectiveness of the probe for UO_2_^2+^ sensing.

## 6. Conclusions

Since the first introduction of fluorescence probes for metal ion sensing, great progress has been achieved both in fundamental mechanism and applications. The rapid development in instrumental tools and dyes not only allows the quantitative measurement of target molecules, but also permits in situ mapping the distribution of the analytes in a spatially resolved manner. In the past years, AIE-based sensors also contribute to the investigation of metal ions in living biosystem. Various fluorescent structures have been designed providing activatable signal and high sensitivity. Further decoration of these AIEgens with recognition moieties brings metal coordination or reaction abilities, thus high selectivity. The biological applications of the probes have been extended from simply qualitative detection to quantitative analysis and real-time tracing of metal ions in samples including living cells, microbe, plants, and fishes. The obtained information can benefit the insights into the roles of these metal ions.

There is broad space remaining for the future development of novel AIE-based sensors for metal ions. Considering the crucial biological functions of Na^+^, K^+^, Ca^2+^, and Mg^2+^, AIE sensors for them are relatively lack. Sensors selectively responding to these essential ions are desirable. Aiming at bioanalytical applications, attentions still should be paid on the water solubility and biocompability of the sensors. Shifting the emission to long wavelength range such as near infrared region is also appealing for bioimaging. This can benefit deep tissue penetrability thus enable monitoring of metal ions inside tissues. Due to the advantages of high penetrability and low phototoxicity, two-photon probes also attract increasing research interests. The development of two-photon AIEgens will benefit the investigation of metal ions in biosystems. Probes allowing tracking spatial distribution and kinetic process of cellular uptake of metal ions are also demanded. It is for sure that AIEgens for metal ions will continuously emerge and contribute to the field of bioanalysis.

## References

[1] Carter K.P., Young A.M., Palmer A.E. (2014). Fluorescent sensors for measuring metal ions in living systems. Chem. Rev..

[2] Bear M.F., Connors B., Paradiso M. (2015). Neuroscience: Exploring the Brain.

[3] Bischof H., Burgstaller S., Waldeck-Weiermair M., Rauter T., Schinagl M., Ramadani-Muja J., Graier W.F., Malli R. (2019). Live-cell imaging of physiologically relevant metal ions using genetically encoded FRET-based probes. Cells.

[4] Nelson D.L., Cox M.M. (2012). Lehninger Principles of Biochemistry.

[5] Weaver R.F. (2013). Molecular Biology.

[6] Rauk A. (2009). The chemistry of Alzheimer’s disease. Chem. Soc. Rev..

[7] Qian X.H., Xu Z.C. (2015). Fluorescence imaging of metal ions implicated in diseases. Chem. Soc. Rev..

[8] Ratte H.T. (1999). Bioaccumulation and toxicity of silver compounds: A review. Environ. Toxicol. Chem..

[9] McLaughlin M.J., Parker D.R., Clarke J.M. (1999). Metals and micronutrients-food safety issues. Field Crop. Res..

[10] Waisberg M., Joseph P., Hale B., Beyersmann D. (2003). Molecular and cellular mechanisms of cadmium carcinogenesis. Toxicology.

[11] de Silva A.P., Gunaratne H.Q.N., Gunnlaugsson T., Huxley A.J.M., McCoy C.P., Rademacher J.T., Rice T.E. (1997). Signaling recognition events with fluorescent sensors and switches. Chem. Rev..

[12] Lee M.H., Kim J.S., Sessler J.L. (2015). Small molecule-based ratiometric fluorescence probes for cations, anions, and biomolecules. Chem. Soc. Rev..

[13] Guo Z.Q., Park S., Yoon J., Shin I. (2014). Recent progress in the development of near-infrared fluorescent probes for bioimaging applications. Chem. Soc. Rev..

[14] Zhou J., Ma H.M. (2016). Design principles of spectroscopic probes for biological applications. Chem. Sci..

[15] Antina E.V., Bumagina N.A., V’yugin A.I., Solomonov A.V. (2017). Fluorescent indicators of metal ions based on dipyrromethene platform. Dye. Pigment..

[16] Chan J., Dodani S.C., Chang C.J. (2012). Reaction-based small-molecule fluorescent probes for chemoselective bioimaging. Nat. Chem..

[17] Jun M.E., Roy B., Ahn K.H. (2011). “Turn-on” fluorescent sensing with “reactive” probes. Chem. Commun..

[18] Johnson I., Spence M.T.Z. (2010). The Molecular Probes Handbook.

[19] Gao M., Tang B.Z. (2017). Fluorescent sensors based on aggregation-induced emission: Recent advances and perspectives. ACS Sens..

[20] Luo J., Xie Z., Lam J.W.Y., Cheng L., Chen H., Qiu C., Kwok H.S., Zhan X., Liu Y., Zhu D. (2001). Aggregation-induced emission of 1-methyl-1,2,3,4,5-pentaphenylsilole. Chem. Commun..

[21] Niu G.L., Zhang R.Y., Gu Y., Wang J.G., Ma C., Kwok R.T.K., Lam J.W.Y., Sung H.H.Y., Williams I.D., Wong K.S. (2019). Highly photostable two-photo NIR AIEgens with tunable organelle specificity and deep tissue penetration. Biomaterials.

[22] Mei J., Leung N.L.C., Kwok R.T.K., Lam J.W.Y., Tang B.Z. (2015). Aggregation-induced emission: Together we shine, united we soar!. Chem. Rev..

[23] Mei J., Hong Y., Lam J.W.Y., Qin A., Tang Y., Tang B.Z. (2014). Aggregation-induced emission: The whole is more brilliant than the parts. Adv. Mater..

[24] Chen J.W., Law C.C.W., Lam J.W.Y., Dong Y.P., Lo S.M.F., Williams I.D., Zhu D.B., Tang B.Z. (2003). Synthesis, light emission, nanoaggregation, and restricted intramolecular rotation of 1,1-substituted 2,3,4,5-tetraphenylsiloles. Chem. Mater..

[25] Cai Y.J., Du L.L., Samedov K., Gu X.G., Qi F., Sung H.H.Y., Patrick B.O., Yan Z.P., Jiang X.F., Zhang H.K. (2018). Deciphering the working mechanism of aggregation-induced emission of tetraphenylethylene derivatives by ultrafast spectroscopy. Chem. Sci..

[26] Zhuang Y., Huang F.J., Xu Q., Zhang M.S., Lou X.D., Xia F. (2016). Facile, fast-responsive, and photostable imaging of telomerase activity in living cells with a fluorescence turn-on manner. Anal. Chem..

[27] Huang Y.Y., Hu F., Zhao R., Zhang G.X., Yang H., Zhang D.Q. (2014). Tetraphenylethylene conjugated with a specific peptide as a fluorescence turn-on bioprobe for the highly specific detection and tracing of tumor markers in live cancer cells. Anal. Chem..

[28] Xu X.J., Huang J., Li J.J., Yan J.W., Qin J.G., Li Z. (2011). A graphene oxide-based AIE biosensor with high selectivity toward bovine serum albumin. Chem. Commun..

[29] Shustova N.B., Ong T.C., Cozzolino A.F., Michaelis V.K., Griffin R.G., Dinca M. (2012). Phenyl ring dynamics in a Tetraphenylethylene-bridged metal-organic framework: Implications for the mechanism of aggregation-induced emission. J. Am. Chem. Soc..

[30] Shi H.B., Liu J.Z., Geng J.L., Tang B.Z., Liu B. (2012). Specific detection of integrin α_v_β_3_ by light-up bioprobe with aggregation-induced emission characteristics. J. Am. Chem. Soc..

[31] Gu X.G., Kwok R.T.K., Lam J.W.Y., Tang B.Z. (2017). AIEgens for biological process monitoring and disease theranostics. Biomaterials.

[32] Hu F., Huang Y.Y., Zhang G.X., Zhao R., Yang H., Zhang D.Q. (2014). Targeted bioimaging and photodynamic therapy of cancer cells with an activatable red fluorescent bioprobe. Anal. Chem..

[33] Xu C.H., Zou H., Zhao Z., Zhang P.F., Kwok R.T.K., Lam J.W.Y., Sung H.H.Y., Williams I.D., Tang B.Z. (2019). A new strategy toward “simple” water-soluble AIE probes for hypoxia detection. Adv. Funct. Mater..

[34] Zhang R.Y., Sung S.H.P., Feng G.X., Zhang C.J., Kenry, Tang B.Z., Liu B. (2018). Aggregation-induced emission probe for specific turn-on quantification of soluble transferrin receptor: An important disease marker for iron deficiency anemia and kidney diseases. Anal. Chem..

[35] Yin J., Hu Y., Yoon J. (2015). Fluorescent probes and bioimaging: Alkali metals, alkaline earth metals and pH. Chem. Soc. Rev..

[36] Lu D., He L., Wang Y., Xiong M., Hu M., Liang H., Huan S., Zhang X.B., Tan W. (2017). Tetraphenylethene derivative modified DNA oligonucleotide for in situ potassium ion detection and imaging in living cells. Talanta.

[37] Liu M., Yu X., Li M., Liao N.X., Bi A.Y., Jiang Y.P., Liu S., Gong Z.C., Zeng W.B. (2018). Fluorescent probes for the detection of magnesium ions (Mg^2+^): From design to application. RSC Adv..

[38] Carroll M.F., Schade D.S. (2003). A practical approach to hypercalcemia. Am. Fam. Physician..

[39] Zaheer A., Murshed M., De Grand A.M., Morgan T.G., Karsenty G., Frangioni J.V. (2006). Optical imaging of hydroxyapatite in the calcified vasculature of transgenic animals. Arterioscler. Thromb. Vasc. Biol..

[40] Bian Y.J., Wang L.Q., Cao F.X., Tang L.J. (2015). A simple fluorescence probe based on aggregation-induced emission (AIE) property for the detection of Mg^2^^+^ ions. J. Fluoresc..

[41] Ishiwari F., Hasebe H., Matsumura S., Hajjaj F., Horii-Hayashi N., Nishi M., Someya T., Fukushima T. (2016). Bioinspired design of a polymer gel sensor for the realization of extracellular Ca^2+^ imaging. Sci. Rep..

[42] Morishima K., Ishiwari F., Matsumura S., Fukushima T., Shibayama M. (2017). Mesoscopic structural aspects of Ca^2+^-triggered polymer chain folding of a tetraphenylethene-appended poly(acrylic acid) in relation to its aggregation-induced emission behavior. Macromolecules.

[43] Gao M., Li Y.X., Chen X.H., Li S.W., Ren L., Tang B.Z. (2018). Aggregation-induced emission probe for light-up and in situ detection of calcium ions at high concentration. ACS Appl. Mater. Interfaces.

[44] Zhang J.D., Yan Z., Wang S., She M.Y., Zhang Z., Cai W.Z., Liu P., Li J.L. (2017). Water soluble chemosensor for Ca^2+^, based on aggregation-induced emission characteristics and its fluorescence imaging in living cells. Dye. Pigment..

[45] Wang P., Jia K., Zhou X., Guan X., Wang L., Tian Y., Wu C., Liu X. (2017). Ca^2+^ induced crosslinking of AIE-active polyarylene ether nitrile into fluorescent polymeric nanoparticles for cellular bioimaging. Macromol. Rapid Commun..

[46] Chen G.L., Zhou Z.C., Feng H., Zhang C.Y., Wang Y.F., Qian Z.S., Pan J.W. (2019). An Aggregation-induced phosphorescence probe for calcium ion-specific detection and live-cell imaging in *Arabidopsis Thaliana*. Chem. Commun..

[47] Rurack K. (2001). Flipping the light switch ‘ON’-the design of sensor molecules that show cation-induced fluorescence enhancement with heavy and transition metal ions. Spectroc. Acta Pt. A-Molec. Biomolec. Spectr..

[48] Aron A.T., Ramos-Torres K.M., Cotruvo J.A., Chang C.J. (2015). Recognition- and reactivity-based fluorescent probes for studying transition metal signaling in living systems. Acc. Chem. Res..

[49] Stein E.D., Cohen Y., Winer A.M. (1996). Environmental distribution and transformation of mercury compounds. Crit. Rev. Environ. Sci. Technol..

[50] Li W.C., Tse H.F. (2015). Health risk and significance of mercury in the environment. Environ. Sci. Pollut. Res..

[51] Wang K., Li J.J., Ji S.M., Li L.J., Qiu Z.P., Pan C.Q., Zhang J.Y., Huo Y.P. (2018). Fluorescence probes based on AIE luminogen: Application for sensing Hg^2+^ in aqueous media and cellular imaging. New J. Chem..

[52] Zhang G.B., Ding A.X., Zhang Y., Yang L.M., Kong L., Zhang X.J., Tao X.T., Tian Y.P., Yang J.X. (2014). Schiff base modified α-cyanostilbene derivative with aggregation-induced emission enhancement characteristics for Hg^2+^ detection. Sens. Actuator B-Chem..

[53] Zhang G.B., Zhang X.J., Zhang Y., Wang H., Kong L., Tian Y.P., Tao X.T., Hong B., Yang J.X. (2015). Design of turn-on fluorescent probe for effective detection of Hg^2+^ by combination of AIEE-active fluorophore and binding site. Sens. Actuator B-Chem..

[54] Fang W.Y., Zhang G.B., Chen J., Kong L., Yang L.M., Bi H., Yang J.X. (2016). An AIE active probe for specific sensing of Hg^2+^ based on linear conjugated bis-Schiff base. Sens. Actuator B-Chem..

[55] Gui S.L., Huang Y.Y., Hu F., Jin Y.L., Zhang G.X., Zhang D.Q., Zhao R. (2018). Bioinspired peptide for imaging Hg^2+^ distribution in living cells and Zebrafish based on coordination-mediated supramolecular assembling. Anal. Chem..

[56] Yuan B., Wang D.X., Zhu L.N., Lan Y.L., Cheng M., Zhang L.M., Chu J.Q., Li X.Z., Kong D.M. (2019). Dinuclear HgII tetracarbene complex-triggered aggregation-induced emission for rapid and selective sensing of Hg^2+^ and organomercury species. Chem. Sci..

[57] Huang L.T., Li S.W., Ling X., Zhang J., Qin A.J., Zhuang J., Gao M., Tang B.Z. (2019). Dual detection of bioaccumulated Hg^2+^ based on luminescent bacteria and aggregation-induced emission. Chem. Commun..

[58] Wu Y.Q., Wen X.Y., Fan Z.F. (2019). An AIE active pyrene based fluorescent probe for selective sensing Hg^2+^ and imaging in live cells. Spectroc. Acta Pt. A-Molec. Biomolec. Spectr..

[59] Bahta M., Ahmed N. (2019). Naphthalimide-amino acid conjugates chemosensors for Hg^2+^ detection: Based on chelation mediated emission enhancement in aqueous solution. J. Photochem. Photobiol. A-Chem..

[60] Gabr M.T., Pigge F.C. (2017). A turn-on AIE active fluorescent sensor for Hg^2+^ by combination of 1,1-bis(2-pyridyl)ethylene and thiophene/bithiophene fragments. Mater. Chem. Front..

[61] Jiang Y., Duan Q.Y., Zheng G.S., Yang L., Zhang J., Wang Y.F., Zhang H.T., He J., Sun H.Y., Ho D. (2019). An ultra-sensitive and ratiometric fluorescent probe based on the DTBET process for Hg^2+^ detection and imaging applications. Analyst.

[62] Mukherjee S., Thilagar P. (2013). Molecular flexibility tuned emission in “V” shaped naphthalimides: Hg(II) detection and aggregation induced emission enhancement (AIEE). Chem. Commun..

[63] Wang A.Z., Yang Y.X., Yu F.F., Xue L.W., Hu B.W., Fan W.P., Dong Y.J. (2015). A highly selective and sensitive fluorescent probe for quantitative detection of Hg^2+^ based on aggregation-induced emission features. Talanta.

[64] Chatterjee A., Banerjee M., Khandare D.G., Gawas R.U., Mascarenhas S.C., Ganguly A., Gupta R., Joshi H. (2017). Aggregation-induced emission-based chemodosimeter approach for selective sensing and imaging of Hg(II) and methylmercury species. Anal. Chem..

[65] Gao T., Huang X.Y., Huang S., Dong J., Yuan K., Feng X.P., Liu T.T., Yu K.Q., Zeng W.B. (2019). Sensitive water-soluble fluorescent probe based on umpolung and aggregation-induced emission strategies for selective detection of Hg^2+^ in living cells and Zebrafish. J. Agric. Food Chem..

[66] Huang S., Gao T., Bi A.Y., Cao X.Z., Feng B., Liu M., Du T., Feng X.P., Zeng W.B. (2020). Revealing aggregation-induced emission effect of imidazolium derivatives and application for detection of Hg^2+^. Dye Pigment..

[67] Chen S., Wang W.J., Yan M.M., Tu Q., Chen S.W., Li T.B., Yuan M.S., Wang J.Y. (2018). 2-Hydroxy benzothiazole modified rhodol: Aggregation-induced emission and dual-channel fluorescence sensing of Hg^2+^ and Ag^+^ ions. Sens. Actuator B-Chem..

[68] Niu C.X., Liu Q.L., Shang Z.H., Zhao L., Ouyang J. (2015). Dual-emission fluorescent sensor based on AIE organic nanoparticles and Au nanoclusters for the detection of mercury and melamine. Nanoscale.

[69] Kepp K.P., Squitti R. (2019). Copper imbalance in Alzheimer’s disease: Convergence of the chemistry and the clinic. Coord. Chem. Rev..

[70] Dujols V., Ford F., Czarnik A.W. (1997). A long-wavelength fluorescent chemodosimeter selective for Cu(II) ion in water. J. Am. Chem. Soc..

[71] Xu J., Hou Y., Ma Q., Wu X., Feng S., Zhang J., Shen Y. (2014). A highly selective fluorescent probe for Cu^2+^ based on rhodamine B derivative. Spectroc. Acta Pt. A-Molec. Biomolec. Spectr..

[72] Wu W.N., Mao P.D., Wang Y., Mao X.J., Xu Z.Q., Xu Z.H., Zhao X.L., Fan Y.C., Hou X.F. (2018). AEE active Schiff base-bearing pyrene unit and further Cu^2+^-induced self-assembly process. Sens. Actuator B-Chem..

[73] Singh A., Singh R., Shellaiah M., Prakash E.C., Chang H.C., Raghunath P., Lin M.C., Lin H.C., Liu B., Zhou H.L. (2017). Aggregation-induced emission activity and further Cu^2+^-induced self-assembly process of two Schiff compounds. Sens. Actuator B-Chem..

[74] Wang Y., Wu H., Wu W.N., Li S.J., Xu Z.H., Xu Z.Q., Fan Y.C., Zhao X.L., Liu B.Z. (2018). An AIRE active Schiff base bearing coumarin and pyrrole unit: Cu^2+^ detection in either solution or aggregation states. Sens. Actuator B-Chem..

[75] Ding A.X., Shi Y.D., Zhang K.X., Sun W., Tan Z.L., Lu Z.L., He L. (2018). Self-assembled aggregation-induced emission micelle (AIE micelle) as interfacial fluorescence probe for sequential recognition of Cu^2+^ and ATP in water. Sens. Actuator B-Chem..

[76] Maret W. (2001). Crosstalk of the group IIa and IIb metals calcium and zinc in cellular signaling. Proc. Natl. Acad. Sci. USA.

[77] Frederickson C.J., Koh J.Y., Bush A.I. (2005). The neurobiology of zinc in health and disease. Nat. Rev. Neurosci..

[78] Arena G., Rizzarelli E. (2019). Zn^2+^ interaction with Amyloid-B: Affinity and speciation. Molecules.

[79] Wang D., Li S.M., Zheng J.Q., Kong D.Y., Zheng X.J., Fang D.C., Jin L.P. (2017). Coordination-directed stacking and aggregation-induced emission enhancement of the Zn(II) Schiff base complex. Inorg. Chem..

[80] Wen X.Y., Wang Q., Fan Z.F. (2018). An active fluorescent probe based on aggregation-induced emission for intracellular bioimaging of Zn^2+^ and tracking of interactions with single-stranded DNA. Anal. Chim. Acta.

[81] Wan J.Y., Zhang W., Guo H.D., Liang J.J., Huang D.Y., Xiao H.B. (2019). Two spirobifluorene-based fluorescent probes with aggregation-induced emission properties: Synthesis and application in the detection of Zn^2+^ and cell imaging. J. Mater. Chem. C.

[82] He X.X., Wang X.M., Zhang L., Fang G.Z., Liu J.F., Wang S. (2018). Sensing and intracellular imaging of Zn^2+^ based on affinity peptide using an aggregation induced emission fluorescence “switch-on” probe. Sens. Actuator B-Chem..

[83] Naskar B., Dhara A., Maiti D.K., Kukulka M., Mitoraj M.P., Srebro-Hooper M., Prodhan C., Chaudhuri K., Goswami S. (2019). Aggregation-induced emission-based sensing platform for selective detection of Zn^2+^: Experimental and theoretical investigations. Chem. Phys. Chem..

[84] Wang J.X., Lin X.F., Shu T., Su L., Liang F., Zhang X.J. (2019). Self-assembly of metal nanoclusters for aggregation-induced emission. Int. J. Mol. Sci..

[85] Guo Y.M., Cao F.P., Lei X.L., Mang L.H., Cheng S.J., Song J.T. (2016). Fluorescent copper nanoparticles: Recent advances in synthesis and applications for sensing metal ions. Nanoscale.

[86] Lin L.Y., Hu Y.F., Zhang L.L., Huang Y., Zhao S.L. (2017). Photoluminescence light-up detection of zinc ion and imaging in living cells based on the aggregation induced emission enhancement of glutathione-capped copper nanoclusters. Biosens. Bioelectron..

[87] Jia X.F., Li J., Wang E.K. (2013). Cu nanoclusters with aggregation induced emission enhancement. Small.

[88] Li D., Chen Z.H., Wan Z.H., Yang T.Z., Wang H., Mei X.F. (2016). One-pot development of water-soluble copper nanoclusters with red emission and aggregation induced fluorescence enhancement. RSC Adv..

[89] Rouault T.A. (2006). The role of iron regulatory proteins in mammalian iron homeostasis and disease. Nat. Chem. Biol..

[90] Gozzelin R., Arosio P. (2016). Iron homeostasis in health and disease. Int. J. Mol. Sci..

[91] Lim B., Baek B., Jang K., Lee N.K., Lee J.H., Lee Y., Kim J., Kang S.W., Park J., Kim S. (2019). Novel turn-on fluorescent biosensors for selective detection of cellular Fe^3+^ in lysosomes: Thiophene as a selectivity-tuning handle for Fe^3+^ sensors. Dye. Pigment..

[92] Yang X.D., Chen X.L., Lu X.D., Yan C.G., Xu Y.K., Hang X.D., Qu J.Q., Liu R.Y. (2016). A highly selective and sensitive fluorescent chemosensor for detection of CN^-^, SO_3_^2^^-^ and Fe^3+^ based on aggregation-induced emission. J. Mater. Chem. C.

[93] Yang D.L., Li F., Luo Z.M., Bao B.Q., Hu Y.L., Weng L.X., Cheng Y.X., Wang L.H. (2016). Conjugated polymer nanoparticles with aggregation induced emission characteristics for intracellular Fe^3+^ sensing. J. Polym. Sci. Part A Polym. Chem..

[94] Yang Y., Wang X.Y., Cui Q.L., Cao Q., Li L.D. (2016). Self-assembly of fluorescent organic nanoparticles for iron(III) sensing and cellular imaging. ACS Appl. Mater. Interfaces.

[95] Sinha N., Stegemann L., Tan T.T.Y., Doltsinis N.L., Strassert C.A., Hahn F.E. (2017). Turn-on fluorescence in Tetra-NHC ligands by rigidification through metal complexation: An alternative to aggregation-induced emission. Angew. Chem. Int. Ed..

[96] Kim N.H., Won M., Kim J.S., Huh Y., Kim D. (2019). A highly sensitive and fast responsive fluorescent probe for detection of Gold(III) ions based on the AIEgen disaggregation. Dye Pigment.

[97] Khan R.I., Ramu A., Pitchumani K. (2018). Design and one-pot synthesis of a novel pyrene based fluorescent sensor for selective “turn on”, naked eye detection of Ni^2+^ ions, and live cell imaging. Sens. Actuator B-Chem..

[98] Gui S.L., Huang Y.Y., Hu F., Jin Y.I., Zhang G.X., Yan L.S., Zhang D.Q., Zhao R. (2015). Fluorescence turn-on chemosensor for highly selective and sensitive detection and bioimaging of Al^3+^ in living cells based on ion-induced aggregation. Anal. Chem..

[99] Xu P.F., Bao Z.Y., Yu C.Y., Qiu Q.Q., Wei M.R., Xi W.B., Qian Z.S., Feng H. (2019). A water-soluble molecular probe with aggregation-induced emission for discriminative detection of Al^3+^ and Pb^2+^ and imaging in seedling root of *Arabidopsis*. Spectroc. Acta Pt. A-Molec. Biomolec. Spectr..

[100] Singh A., Singh R., Shellaiah M., Prakash E.C., Chang H.C., Raghunath P., Lin M.C., Lin H.C. (2015). A new pyrene-based aggregation induced ratiometric emission probe for selective detections of trivalent metal ions and its living cell application. Sens. Actuator B-Chem..

[101] Simon T., Shellaiah M., Srinivasadesikan V., Lin C.C., Ko F.H., Sun K.W., Lin M.C. (2016). A simple pyrene based AIEE active schiff base probe for selective naked eye and fluorescence off-on detection of trivalent cations with live cell application. Sens. Actuator B-Chem..

[102] Shellaiah M., Simon T., Srinivasadesikan V., Lin C.M., Sun K.W., Ko F.H., Lin M.C., Lin H.C. (2016). Novel pyrene containing monomeric and dimeric supramolecular AIEE active nano-probes utilized in selective ‘’off-on’’ trivalent metal and highly acidic pH sensing with live cell applications. J. Mater. Chem. C.

[103] Gui S.L., Huang Y.Y., Zhu Y.Y., Jin Y.L., Zhao R. (2019). Biomimetic sensing system for tracing Pb^2+^ distribution in living cells based on the metal-peptide supramolecular assembly. ACS Appl. Mater. Interfaces.

[104] Chen X.T., Peng L., Feng M.L., Xiang Y., Tong A.J., He L.F., Liu B., Tang Y.P. (2017). An aggregation induced emission enhancement-based ratiometric fluorescent sensor for detecting trace uranyl ion (UO_2_^2+^) and the application in living cells imaging. J. Lumines..

